# Epidemic waves caused by SARS‐CoV‐2 omicron (B.1.1.529) and pessimistic forecasts of the COVID‐19 pandemic duration

**DOI:** 10.1002/mco2.122

**Published:** 2022-03-09

**Authors:** Igor Nesteruk

**Affiliations:** ^1^ Institute of Hydromechanics National Academy of Sciences of Ukraine Kyiv Ukraine

Dear editor,

The sharp increase in the number of new COVID‐19 cases in late 2021 and early 2022 associated with the SARS‐CoV‐2 omicron (B.1.1.529) strain made it necessary to update the forecasts. Comparative analysis yielded some preliminary predictions of time durations when the maximal number of new cases are expected.^1^ To estimate the duration of new pandemic waves and the number of infectious persons, different versions of susceptible‐infected‐removed (SIR) model can be applied (see e.g.,^2^, ^3^, ^4^). In particular, generalized SIR model and a corresponding parameter identification procedure^4^ were used to simulate 13 epidemic waves for Ukraine and six pandemic waves for the whole world. Thus, we can hope for an accurate forecast for the omicron waves (14th in Ukraine and seventh in the world), to which this study is devoted.

We will use the dataset regarding the accumulated numbers of laboratory‐confirmed COVID‐19 cases *V_j_* in Ukraine and the whole world from the COVID‐19 Data Repository by the Center for Systems Science and Engineering (CSSE) at Johns Hopkins University (JHU)^5^ (Tables S1 and S2). To simulate the 14th wave in Ukraine and seventh wave in the world, we will use the datasets, corresponding to the period January 22 to February 4, 2022. Other values of *V_j_* and corresponding moments of time *t_j_* (measured in days) will be used to control the accuracy of calculations only.

The generalized SIR model relates the number of susceptible *S*(*t*), infectious *I*(*t*), and removed persons *R*(*t*) versus time *t* for a particular epidemic wave *i*.^4^ The exact solution of the set of nonlinear differential equations uses the function V(t)=I(t)+R(t), corresponding to the number of victims or the cumulative laboratory‐confirmed number of cases (Supporting Methods^4^). Its derivative *dV*/*dt* yields the estimation of the average daily number of new cases. When the registered number of victims *V_j_* is a random realization of its theoretical dependence, the exact solution presented in the study by Nesteruk^4^ depends on five parameters (see Supporting Materials). The details of the optimization procedure for their identification can be found in.^4^ As daily numbers of new cases are random, we will use the smoothed number of accumulated cases and its numerical derivative to estimate the smoothed number of new daily cases (see details in the study by Nesteruk^4^).

The optimal values of parameters and SIR curves are shown in Figure 1. Black lines correspond to the 14th wave in Ukraine, purple ones to the seventh wave in the whole world (Figure 1A). The optimal values of SIR parameters for previous pandemic waves are shown in Figure 1B for comparison. “Stars” and “crosses” in Figure 1A illustrate the accuracy of simulations for the accumulated number of cases and the averaged daily number of new cases. Comparisons with corresponding black solid and dotted lines in Figure 1B show that the theoretical estimations for the 14th wave in Ukraine were consistent with observations after January 7, 2022 (see black markers). The accuracy of SIR simulations for the global seventh wave is also very good (compare purple dotted and solid lines with purple markers in Figure 1A).

**FIGURE 1:**
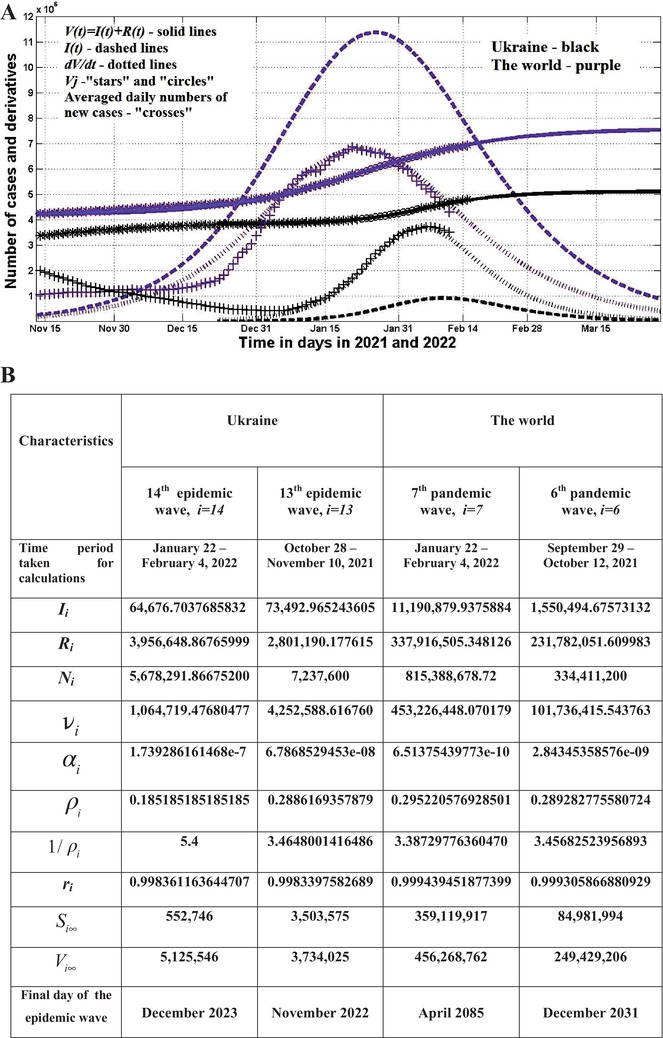
The omicron waves in Ukraine and in the whole world. A, The results of susceptible‐infected‐removed (SIR) simulations of the 14th wave in Ukraine are shown by black lines. Purple lines represent the seventh pandemic wave in the world. Number of victims *V*(*t*)* = I*(*t*)* + R*(*t*) – solid lines (for the world divided by 60); numbers of infected and spreading *I*(*t*) (multiplied by 5 for Ukraine) – dashed; derivatives *dV*/*dt*, (Equation 7, multiplied by 100 for Ukraine and by 2 for the world) – dotted. “Circles” correspond to the accumulated numbers of cases registered during the periods of time taken for SIR simulations (for the world divided by 60). “Stars” correspond to *V_j_* values beyond these time periods (for the world divided by 60). “Crosses” show the numerical first derivative (14) multiplied by 100 for Ukraine and by 2 for the world. Black markers correspond to Ukraine and purple to the world (according to JHU datasets,^5^ Tables S1 and S2). B, Optimal values of SIR parameters and other pandemic characteristics

According to the predictions for the 14th wave in Ukraine, the average daily numbers of new cases and infectious persons will stop to increase around February 6 and 10, 2022, respectively (see black dotted and dashed lines in Figure 1A). The day with the maximum of new cases for the omicron wave in Ukraine correlates with the results of comparative analysis presented by Nesteruk and Rodionov^1^ and recent observations shown by black crosses.

The assessments of the pandemic wave durations (corresponding the moment when the number of infectious persons will be less that unit) become more and more pessimistic (see Figure 1B). There is no hope of a rapid decline of omicron waves. For Ukraine, the end of the 14th wave is expected in December 2023. The day when the number of infected persons in Ukraine will be less than 10 corresponds to July 19, 2022.

One could expect full control of the epidemic in Ukraine in the summer of 2022. But this would only be possible in complete isolation and in the absence of new resistant strains that could cause new waves. Unfortunately, the emergence of new variants of coronavirus is almost unpredictable, but the presence of a large number of patients in the world increases its likelihood. According to our estimations, at the end of March 2022, the number of infectious people in the world will be around 1 million (see the purple dashed line in Figure 1A). This fact and the absence of isolation reduce the optimism about the forecast for the summer of 2022 for Ukraine and Europe.

Calculated duration of the SARS‐CoV‐2 wave in the world is very long (in more than 63 years, the number of infectious persons will be less than one and in 6.7 years less than 10). This result can be explained by the huge number of people spreading the infection (see the dashed blue line in Figure 1A). These facts let us to conclude that if the situation with vaccination, testing, and treatment will not change, humanity may be forced to live with the coronavirus forever.

The good news is the decline in mortality rate for the omicron wave compared to the previous one. As of February 1, 2022, the deaths per case ratios were 0.0049 and 0.0032 for Ukraine and the world, respectively (to calculate these figures we have used the averaged daily values of new deaths and cases reported by JHU^5^). For the days corresponding to the averaged daily maximum of new cases in the previous waves, the deaths per case ratios were approximately five times higher (0.0253: Ukraine November 1, 2021; 0.0153: the world, August 24, 2021). It can be connected with the easier course of the disease caused by omicron and with the increase in the vaccination level (the percentage of fully vaccinated people has increased from 17.3% to 33.8% for Ukraine and from 24.7% to 51.9% for the world^5^). To identify the influence of both factors could help a special statistical analysis. But we can already see the threatening trend of rising mortality rate in South Africa (0.0397: February 1, 2022, vaccination level 27.7%; 0.0013: December 10, 2021, vaccination level 25.5%^5^).

In summary, the generalized SIR model and corresponding parameter identification procedure were used to simulate and predict the dynamics of omicron waves in Ukraine and in the whole world. Results of calculations yield very long duration of the COVID‐19 epidemic (more than 63 years). If the global situation with quarantine restrictions, vaccination, testing, and treatment will not change, humanity may be forced to live with the coronavirus forever.

## CONFLICT OF INTEREST

The author declares that there is no conflict of interest.

## ETHICS APPROVAL

Not applicable.

## FUNDING INFORMATION

The study was not supported by any funding.

## Supporting information



Supporting Information

## Data Availability

The data included in this study are available upon request from the corresponding author.
