# Supplementary material for: Epidemic waves caused by SARS‐CoV‐2 omicron (B.1.1.529) and pessimistic forecasts of the COVID‐19 pandemic duration
Source: MedComm (2020). 2022 Mar 9;3(1):e122. doi: 10.1002/mco2.122 (PMC8906450; doi:10.1002/mco2.122)
Supplement: Supplementary file 1 — Supporting Information [file MCO2-3-0-s001.doc]

**Epidemic waves caused by SARS-CoV-2 Omicron (B.1.1.529) and pessimistic forecasts of the COVID-19 pandemic duration**

**Supplementary**

**Table S1. Cumulative numbers of laboratory-confirmed Covid-19 cases in Ukraine for the period of November 1, 2021 to February 15, 2022 according to JHU report on February 16, 2022, [1].**

| Day in corres-ponding month of 2021 and 2022 | Number of cases in November  2021,  *V_j_* | Number of cases  in December  2021,  *V_j_* | Number of cases  in January  2022,  *V_j_* | Number of cases  in February 2022,  *V_j_* |
| --- | --- | --- | --- | --- |
| 1 | 3073125 | 3619223 | 3852397 | 4287117 |
| 2 | 3093661 | 3633386 | 3854405 | 4323009 |
| 3 | 3118140 | 3647777 | 3856359 | 4363754 |
| 4 | 3146617 | 3661583 | 3858248 | 4408776 |
| 5 | 3174223 | 3668794 | 3862959 | 4452612 |
| 6 | 3200411 | 3673839 | 3869728 | 4481918 |
| 7 | 3218967 | 3683044 | 3877032 | 4506669 |
| 8 | 3233178 | 3692939 | 3880371 | 4542568 |
| 9 | 3253327 | 3705823 | 3883316 | 4582137 |
| 10 | 3277772 | 3717640 | 3885416 | 4625614 |
| 11 | 3303694 | 3728246 | 3890974 | 4668581 |
| 12 | 3328934 | 3733967 | 3898240 | 4708604 |
| 13 | 3353694 | 3738390 | 3908469 | 4735258 |
| 14 | 3369387 | 3746106 | 3919151 | 4753922 |
| 15 | 3381399 | 3754567 | 3929950 | 4785138 |
| 16 | 3398913 | 3764485 | 3936582 |  |
| 17 | 3418792 | 3773700 | 3941923 |  |
| 18 | 3440602 | 3781506 | 3950774 |  |
| 19 | 3461873 | 3785395 | 3963917 |  |
| 20 | 3481347 | 3788209 | 3982738 |  |
| 21 | 3493203 | 3794490 | 4003280 |  |
| 22 | 3501815 | 3801079 | 4026198 |  |
| 23 | 3515641 | 3808612 | 4042152 |  |
| 24 | 3530969 | 3815440 | 4055643 |  |
| 25 | 3548842 | 3820891 | 4075351 |  |
| 26 | 3565644 | 3823879 | 4100292 |  |
| 27 | 3580671 | 3825917 | 4133396 |  |
| 28 | 3588916 | 3828336 | 4168560 |  |
| 29 | 3595410 | 3833952 | 4206731 |  |
| 30 | 3606622 | 3840041 | 4232143 |  |
| 31 | - | 3847226 | 4255206 |  |

**Table S2. Cumulative numbers of laboratory-confirmed Covid-19 cases in the world for the period of November 1, 2021 to February 15, 2022 according to JHU report on February 16, 2022, [1].**

| Day in corres-ponding month of 2021 and 2022 | Number of cases in November 2021,  *V_j_* | Number of cases in December  2021,  *V_j_* | Number of cases  in January  2022,  *V_j_* | Number of cases  in February 2022,  *V_j_* |
| --- | --- | --- | --- | --- |
| 1 | 247616683 | 263574605 | 289514753 | 381740417 |
| 2 | 248038986 | 264280433 | 290458926 | 384910273 |
| 3 | 248569605 | 264994581 | 292883351 | 388048849 |
| 4 | 249090238 | 265506199 | 295401488 | 390961200 |
| 5 | 249606778 | 265946530 | 297962232 | 393688512 |
| 6 | 250033256 | 266542652 | 300587060 | 395523030 |
| 7 | 250381000 | 267238222 | 303518939 | 397761141 |
| 8 | 250857194 | 267906078 | 305654158 | 400890606 |
| 9 | 251342787 | 268634462 | 307671496 | 403339453 |
| 10 | 251922291 | 269321177 | 310834537 | 406106920 |
| 11 | 252441695 | 269819944 | 313713290 | 408498744 |
| 12 | 253037119 | 270263297 | 317419339 | 410330280 |
| 13 | 253465760 | 270889422 | 320309661 | 411784471 |
| 14 | 253820659 | 271547009 | 323887251 | 413537270 |
| 15 | 254359567 | 272287381 | 326318495 | 415198318 |
| 16 | 254884883 | 273024840 | 328445640 |  |
| 17 | 255513523 | 273756639 | 331181553 |  |
| 18 | 256127633 | 274336995 | 334915183 |  |
| 19 | 256744664 | 274818834 | 339134127 |  |
| 20 | 257226649 | 275566222 | 342795496 |  |
| 21 | 257626179 | 276357147 | 346636145 |  |
| 22 | 258246323 | 277258354 | 349320573 |  |
| 23 | 258856453 | 278248864 | 351725833 |  |
| 24 | 259529042 | 279093884 | 355209424 |  |
| 25 | 260124955 | 279777852 | 358930099 |  |
| 26 | 260720377 | 280375131 | 362694520 |  |
| 27 | 261179145 | 281659093 | 366365124 |  |
| 28 | 261587226 | 282978871 | 369988033 |  |
| 29 | 262242291 | 284696526 | 372594363 |  |
| 30 | 262868430 | 286633993 | 374815359 |  |
| 31 |  | 288335323 | 378433295 |  |

**Methods of the study**

The classical SIR model for an infectious disease [2-4] was generalized in [5] in order to simulate different epidemic waves. We suppose that the SIR model parameters are constant for every epidemic wave, i.e. for the time periods: $t_{i}^{*}\leq t\leq t_{i+1}^{*}, i=1,2,3,...$. Than for every wave we can use the equations, similar to [2-4]:

$\frac{dS}{dt}=-\alpha_{i}SI$ , (1)

$\frac{dI}{dt}=\alpha_{i}SI-\rho_{i}I$, (2)

$\frac{dR}{dt}=\rho_{i}I$ . (3)

Here *S* is the number of susceptible persons (who are sensitive to the pathogen and **not protected**); *I* is the number of infected persons (who are sick and **spread the infection**; please don’t confuse with the number of still ill persons, so known active cases) and *R* is the number of removed persons (who **no longer spread the infection**; this number is the sum of isolated, recovered, dead, and infected people who left the region). Parameters $\alpha_{i}$ and $\rho_{i}$ are supposed to be constant for every epidemic wave.

To determine the initial conditions for the set of equations (1)–(3), let us suppose that at the beginning of every epidemic wave $t_{i}^{*}$:

$I(t_{i}^{*})=I_{i}$, $R(t_{i}^{*})=R_{i}$, $S(t_{i}^{*})=N_{i}-I_{i}-R_{i}$; $N_{i}=S+I+R$ (4)

The corresponding number of susceptible persons at infinity $S_{i\infty}>0$ can be calculated from a non-linear equation

$S_{i\infty}=(N_{i}-I_{i}-R_{i})e^{\frac{S_{i\infty}-N_{i}-R_{i}}{\nu}}$ , $\nu_{i}=\frac{\rho_{i}}{\alpha_{i}}$ (5)

In [5] the set of differential equations (1)-(3) was solved by introducing the function

$V(t)=I(t)+R(t)$, (6)

corresponding to the number of victims or the cumulative confirmed number of cases. For many epidemics (including the COVID-9 pandemic) we cannot observe dependencies $S(t),I(t)$ and $R(t)$ but observations of the accumulated number of cases *V_j_* corresponding to the moments of time *t_j_* provide information for direct assessments of the dependence $V(t)$.

It follows from (2) and (3) that:

$\frac{dV}{dt}=\alpha_{i}SI$ (7)

Then

$\frac{dV}{dt}=\alpha_{i}(N_{i}-V)\left[\nu_{i}\ln(N_{i}-V)+V-R_{i}-\nu_{i}\ln(N_{i}-R_{i}-I_{i})\right]$ (8)

Integration of (8) provides an analytical solution for the set of equations (1)–(3):

$F_{i}^{*}(V,N_{i},I_{i},R_{i},\nu_{i})=\alpha_{i}(t-t_{i}^{*})$, (9)

$F_{i}^{*}=\int_{R_{i}+I_{i}}^{V} \frac{dU}{(N-U)\left[\nu_{i}\ln(N_{i}-U)+U-R_{i}-\nu_{i}\ln(N_{i}-R_{i}-I_{i})\right]}$. (10)

Thus, for every set of parameters $N_{i},I_{i},R_{i},\nu_{i},\alpha_{i}$ and a fixed value of $V$, integral (10) can be calculated and the corresponding moment of time can be determined from (9). Then functions *I(t)* and *R(t)*  can be easily calculated with the use of formulas available in [5]. The final numbers of victims (final accumulated number of cases corresponding to the *i-th* epidemic wave) can be calculated from:

$V_{i\infty}=N_{i}-S_{i\infty}$. (11)

To estimate the final day of the *i-th* epidemic wave, we can use the condition:

$I(t_{if})$*=*1. (12)

which means that at $t>t_{if}$ less than one person still spreads the infection.

In the case of a new epidemic, the values of its parameters are unknown and must be identified with the use of limited data sets. The exact solution (9)-(10) depend on five parameters -$N_{i},I_{i},R_{i},\nu_{i},\alpha_{i}$. Then the registered number of victims *V_j_* corresponding to the moments of time *t_j_* can be used in eq. (10) in order to calculate $F_{i,j}=F_{i}^{*}(V_{j},N_{i},\nu_{i},I_{i},R_{i})$ for every fixed values of $N_{i},\nu_{i},I_{i},R_{i}$ and then to check how the registered points fit the straight line (9) with the use of linear regression. Optimal values of parameters correspond to the maximum of the correlation coefficient *r_i_* (see [6]). With the use of this approach thirteen COVID-19 epidemic waves for Ukraine and six waves for the whole world were simulated in [5, 7-9]. A very long wave caused by delta variant was predicted for India [10].

Since daily numbers of new cases are random and characterized by some weekly periodicity, we will use the smoothed daily number of accumulated cases:

$\bar{V}_{i}=\frac{1}{7}\sum_{j=i-3}^{j=i+3} V_{j}$, (13)

and its numerical derivative:

$\left.\frac{d\bar{V}}{dt}\right|_{t=t_{i}}\approx\frac{1}{2}\left(\bar{V}_{i+1}-\bar{V}_{i-1}\right)$ (14)

to estimate the smoothed number of new daily cases [5, 7-9].

**References**

1. [COVID-19 Data Repository by the Center for Systems Science and Engineering (CSSE) at Johns Hopkins University](https://github.com/CSSEGISandData/COVID-19) (JHU). <https://github.com/owid/covid-19-data/tree/master/public/data>. Accessed on February 16, 2022.

2. Kermack WO, McKendrick AG. A Contribution to the mathematical theory of epidemics. J Royal Stat Soc Ser A. 1927;115:700-21.

3. Murray JD. Mathematical Biology I/II. New York: Springer; 2002.

4. Langemann D, Nesteruk I, Prestin J. Comparison of mathematical models for the dynamics of the Chernivtsi children disease. Mathematics in Computers and Simulation. 2016;123:68-79.

5. Nesteruk I. Visible and real sizes of new COVID-19 pandemic waves in Ukraine Innov Biosyst Bioeng. 2021; 5 (2): 85–96.

## 6. Nesteruk I. Statistics based models for the dynamics of Chernivtsi children disease. Naukovi Visti NTUU KPI. 2017;5:26-34.

7. Nesteruk I. Detections and SIR simulations of the COVID-19 pandemic waves in Ukraine. Comput. Math. Biophys. 2021;9:46–65.

8. Nesteruk I. Influence of Possible Natural and Artificial Collective Immunity on New COVID-19 Pandemic Waves in Ukraine and Israel. Explor Res Hypothesis Med. Published online: Nov 11, 2021. doi: 10.14218/ERHM.2021.00044.

9. Nesteruk I. Final sizes and durations of new COVID-19 pandemic waves in Ukraine and around the world predicted by generalized SIR model. MedRxiv. Posted November 24, 2021.https://doi.org/10.1101/2021.11.22.21266683

10. Nesteruk I. The COVID-19 pandemic dynamic in India in the spring and summer of 2021. J Bio Med Open Access. 2021;2(2):123.
